# Rapid Generation and Analysis of a Barley Doubled Haploid Line with Higher Nitrogen Use Efficiency Than Parental Lines by F1 Microspore Embryogenesis

**DOI:** 10.3390/plants10081588

**Published:** 2021-08-01

**Authors:** Hongwei Xu, Yingbo Li, Runhong Gao, Rugen Xu, Guimei Guo, Ruiju Lu, Nigel G. Halford, Zhiwei Chen, Chenghong Liu

**Affiliations:** 1 Shanghai Academy of Agricultural Sciences/Key Laboratory of Agricultural Genetics and Breeding, Biotech Research Institute, Shanghai 201106, China; xuhongwei@saas.sh.cn (H.X.); liyingbo@saas.sh.cn (Y.L.); gaorunhong@saas.sh.cn (R.G.); guoguimei@saas.sh.cn (G.G.); cs7@saas.sh.cn (R.L.); 2 Jiangsu Key Laboratory of Crop Genetics and Physiology/Co-Innovation Center for Modern Production Technology of Grain Crops, Key Laboratory of Plant Functional Genomics of the Ministry of Education, Barley Research Institution of Yangzhou University, Yangzhou University, Yangzhou 225009, China; rgxu@yzu.edu.cn; 3 Department of Plant Sciences, Rothamsted Research, Harpenden AL5 2JQ, UK; nigel.halford@rothamsted.ac.uk

**Keywords:** barley, microspore, doubled haploid line, nitrogen use efficiency, RNA-seq

## Abstract

Creating varieties with high nitrogen use efficiency (NUE) is crucial for sustainable agriculture development. In this study, a superior barley doubled haploid line (named DH45) with improved NUE was produced via F_1_ microspore embryogenesis with three rounds of screening in different nitrogen levels by hydroponic and field experiments. The molecular mechanisms responsible for the NUE of DH45 surpassing that of its parents were investigated by RNA-seq analysis. A total of 1027 differentially expressed genes (DEGs) were identified that were up- or down-regulated in DH45 under low nitrogen conditions but showed no significant differences in the parents. GO analysis indicated that genes involved in nitrogen compound metabolic processes were significantly enriched in DH45 compared with the parents. KEGG analysis showed the MAPK signaling pathway plant to be highly enriched in DH45 relative to its parents, as well as genes involved in alanine, aspartate and glutamate metabolism, and arginine biosynthesis. In conclusion, our study revealed the potential to fix trait superiority in a line by combining crossing with F_1_ microspore culture technologies in future crop breeding and also identified several candidate genes that are expressed in shoots and may enable barley to cope with low-nitrogen stress.

## 1. Introduction

Global food production faces a great challenge to meet the demand of the increasing world population, which is predicted to rise to 9 billion people by 2050 [1,2]. Further, it has been reported that crop yields have either stagnated or collapsed during the period 1961–2008 in 24–39% of the growing areas for maize (*Zea mays*), rice (*Oryza sativa*), wheat (*Triticum aestivum*) and soybean (*Glycine max*) [3]. Nitrogen fertilizer is the most important macronutrient supplied to crops to maintain high yields in most regions [4]. However, it is estimated that more than half of the supplied nitrogen is not absorbed by plants but instead is lost to the environment, causing serious environmental problems, such as ecosystem pollution and climate change, while increasing energy costs [5,6]. It has been recognized that breeding new cultivars with high nitrogen use efficiency (NUE) would be an effective way to increase crop yields under low nitrogen (LN) input for the sustainable development of agriculture [7]. 

Conventional hybrid breeding has been widely used for genetic improvement in crops and has been applied successfully to improve traits such as growth rate, grain yield, and NUE [8,9]. Nevertheless, a major disadvantage of traditional hybridization lies in the subsequent development of homozygous lines, which requires 6–10 generations of inbreeding by selfing or sib-crossing [10]. Doubled haploid (DH) technology provides a valuable adjunct to plant breeding programs, as it enables the rapid generation of completely homozygous lines [11]. It has been widely used in quantitative genetics research to discover recessive, dominant, and deleterious mutations [12,13]. 

In DH breeding technologies, anther culture is often used to produce DH plants; however, isolated microspore culture could serve as a more efficient system [14]. Microspores are immature precursors to pollen grains, which are not interfered with by heterozygous diploid somatic cells from anther wall tissues and also have higher embryogenesis efficiency [10]. Thus far, although there has been some work on microspore mutagenesis breeding technology for improving NUE [15], little attention has been paid to applying crossing combined with isolated F1 microspore culture technology to rapidly generate DH lines with higher NUE than their parents. In comparison with the offspring from conventional hybridization, the progeny of DH lines are more stable and reliable for NUE evaluation. Further, without the influence of heterozygous genes, the superiority imparted by gene stacking from biparental crosses could be fixed in the homozygous state in the DH lines, providing suitable material for profiling specific gene expression patterns [10,11].

Barley (*Hordeum vulgare* L.) is the world’s fourth most important cereal crop after wheat, rice, and maize, and it has been recognized as an excellent model plant for microspore embryogenesis and functional food research [15,16,17,18]. The objective of this study was to rapidly generate barley DH lines with higher nitrogen use efficiency than parental lines by F1 microspore embryogenesis and explore the molecular mechanism for improving NUE by genome-wide transcriptomic profiling.

## 2. Results

### 2.1. DH Lines Produced by a Cross Combined with F1 Isolated Microspore Culture

**Figure 1:**
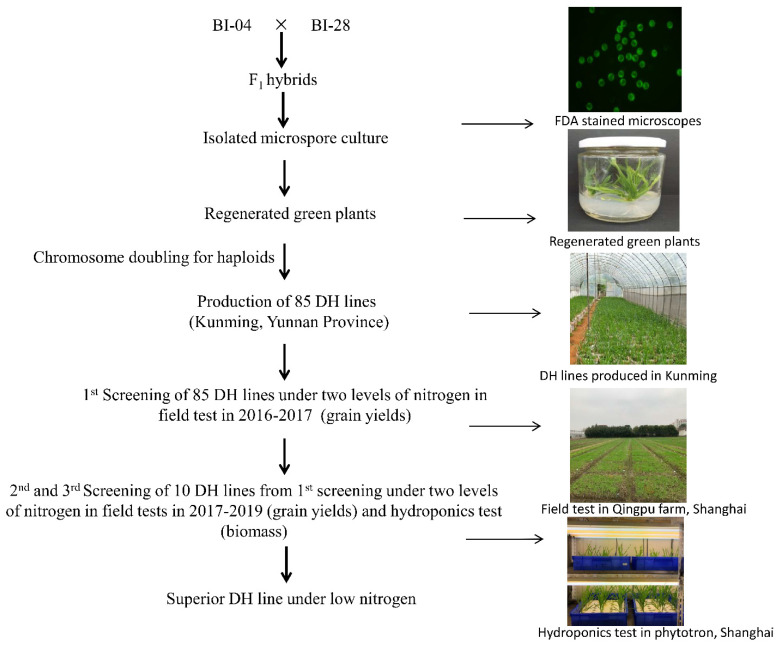
Breeding scheme for the production of super DH line.

The NUE performance of four varieties of barley that are cultivated in the Yangtze River Delta region of China, BI04, BI28, BI35, and BI45, is shown in Table 1. NUE was measured under two nitrogen levels, control (CK; 7.5 mM) and low nitrogen (LN; 0.75 mM), at the seedling stage. The NUE under the two conditions, and hence the relative value of NUE (NUE LN/NUE CK), showed no significant difference between BI04 and BI28 (Table 1). BI04 and BI28 were crossed, and 85 DH lines were produced from F_1_ isolated microspore cultures. DH45, a line with more stable and higher NUE than either parent, was identified from the population growing under the two levels of nitrogen in the hydroponic cultures and field experiments from 2016–2019. The breeding scheme for the production of this ‘super’ DH line is shown in Figure 1.

### 2.2. Identification of DH45 by Screening the DH Population for Lines with Higher NUE Than the Parents in Field Experiments

**Figure 2:**
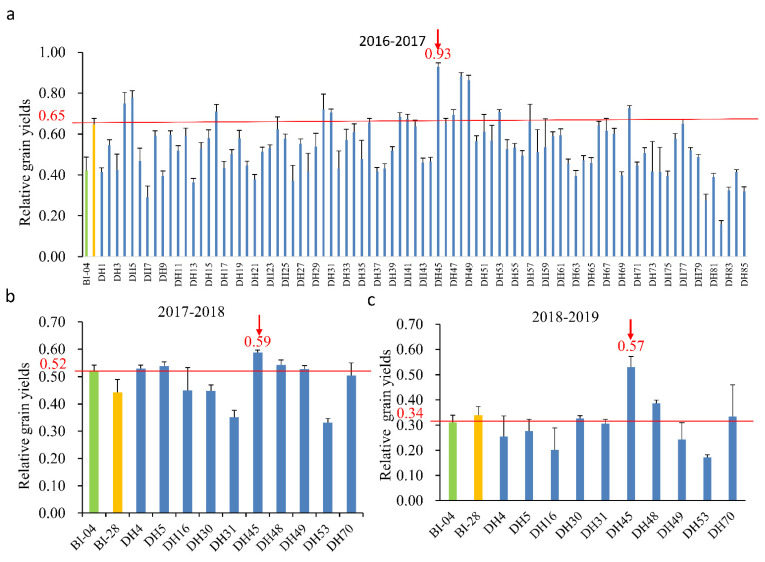
Comparison of relative grain yields between parents and DH lines from the year of 2016 to 2019. (**a**). The 1st screening of 85 DH lines in 2016–2017; (**b**). The 2nd screening of 10 DH lines in 2017–2018; (**c**). The 3rd screening of 10 DH lines in 2018–2019. The green and yellow columns represent the values of BI04 and BI28, respectively. The red lines indicate the value for a better parental line, and the maximum value for a “better” parent was also presented. The arrows indicate the performance of DH45. The relative grain yields were calculated as grain yield (g) under the low nitrogen (LN) condition/grain yield (g) under the control condition (CK). All data represent four biological replicates.

To identify DH lines that were superior to both parents with respect to NUE, the DH population was grown in a field experiment under two levels of nitrogen (150 kg·ha^−1^ and 45 kg·ha^−1^ N applied to soil) over three years (2016–2019). First, 10 DH lines were selected from the 85 DH lines in the population based on having higher relative grain yields than the parents in the first round of screening from 2016 to 2017 (Figure 2a). In the following two rounds of screening, DH45 was selected from these 10 DH lines based on its relative grain yields in field tests from 2017 to 2019 (Figure 2b,c). DH45 exhibited significantly higher NUE than its parents in all growing seasons (73.83 % increase over BI04 and 43.0% over BI28, on average). The grain yield of the DH45 line was consistently higher than that of its parents under low-nitrogen stress, and the difference was significant in two of the three growing seasons (Table 2). These results indicate that DH45 has improved NUE relative to both parents under LN conditions.

### 2.3. Comparing the NUE of DH45 and Its Parents in Hydroponics Experiments

**Figure 3:**
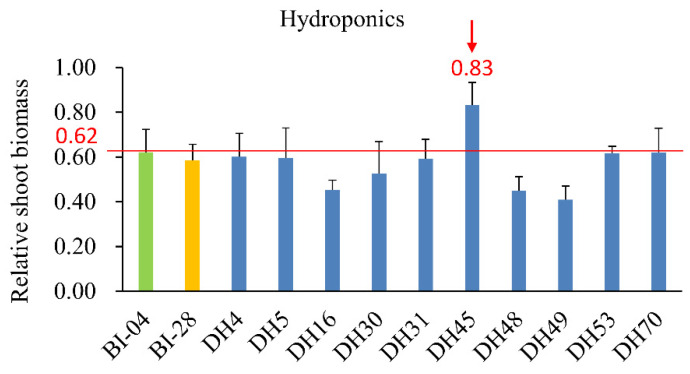
Relative shoot biomass of 7d seedlings of 10 DH lines growing under LN conditions in hydroponics. The green and yellow columns represent the value of BI04 and BI28, respectively. The red lines indicate the value for a better parental line and the maximum value for a “better” parent was also presented. The arrows indicate the performance of DH45. The relative shoot biomass was calculated as shoot biomass under the low-nitrogen (LN) condition/shoot biomass under the control condition (CK). The arrows indicate the performance of DH45. All data represent three biological replicates.

**Figure 4:**
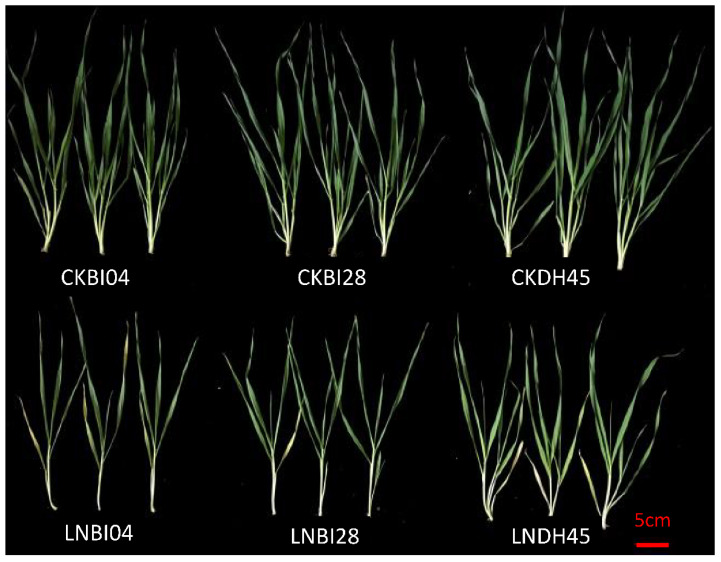
Phenotypic comparison of DH45 and its parents at 28d seedling stage under CK and LN conditions.

The 10 selected DH lines were grown in hydroponics so that nitrogen supply could be closely controlled. DH45 showed a higher relative shoot biomass than that of the parents after 7 d of treatment (Figure 3). Seedlings of DH45 also exhibited phenotypic differences from its parents in the above-ground part of the plant after 28d under LN treatment (0.75 mM N) (Figure 4). Although LN treatment caused a significant reduction in shoot biomass in DH45 and its parents compared with the CK treatment (7.5 mM N), DH45 was much less affected than BI04 and BI28, with 30.77% reduction compared with 60.00% in BI04 and 55.56% in BI28. Consequently, the biomass of DH45 under the LN treatment was 92.86% higher than that of BI04 and 68.75% higher than that of BI28. Sharp increases for NUE were observed in DH45 and its parents under the LN treatment compared with the CK treatment, but the NUE of DH45 under LN conditions was significantly higher than that of either parent (64.65% higher than BI28 and 91.59% higher than BI04). The over middle parent (MPD) and over best parent (HPD) values for DH45 were significant (*p* < 0.05) for both biomass and NUE under LN stress (Table 3). 

### 2.4. Transcriptomic Profiling of DH45 and its Parents

To explore the molecular mechanisms underlying the higher NUE of DH45 compared with its parents under LN conditions, seedlings of DH45 and its parents were grown in hydroponic culture for 7d, and RNA was sampled and analyzed by RNA-seq. The RNA integrity number of samples used for sequencing were all good (Table S1). Principal component analysis (*PCA*) exhibited good quality and repeatability of each biological replicate (Figure S1). On average, 131,392,418 and 128,982,233 clean reads were obtained from three CK samples and three LN treated samples, respectively. The percentage of phred scores at the Q30 level ranged from 90.63 to 93.46%, and the proportion of filtered reads was over 99% (Table S2). In addition, 94.35–95.64% of the reads were mapped to the barley reference genome, of which 4.36–5.65% mapped to intergenic regions and 98.91–99.39% mapped to exons (Table S3). 

### 2.5. Identification of Specific Differentially Expressed Genes (DEGs) in DH45 under LN Conditions 

**Figure 5:**
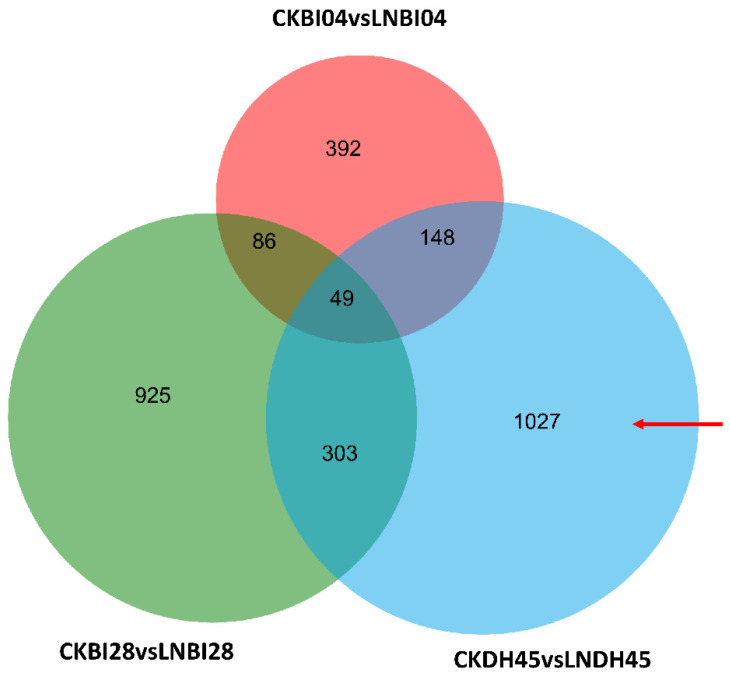
Venn diagrams of DEGs between line DH45 and its parents. The red arrow represents the DEGs specific to DH45; CK vs. LN represents the unique DEGs under the LN condition relative to the CK condition.

DEGs were identified by comparing the data for DH45 and its parents under the LN and CK treatments. A total of 1027 genes were shown to be differentially expressed in DH45 but not in the parents, of which 460 were up-regulated in the LN condition and 567 down-regulated (red arrow in Figure 5). In addition, there were 49 DEGs in common between DH45 and both parents, 303 in common between DH45 and BI28, and 148 in common between DH45 and BI04 (Figure 5). The 1027 DEGs specific to DH45 were categorized as differentially expressed between the hybrid and parents (DEG_HP_) and were selected for further analysis. The up-and down-regulated DEGs are also presented in Figure S2.

### 2.6. Functional Classification of the 1027 DEG_HP_ in DH45 by GO and KEGG Enrichment Analysis 

To ascertain the functional classes of the 1027 DEG_HP_ in DH45, GO functional classification was performed. GO enrichment analysis found that the biological process ontology of “protein phosphorylation” (GO:0006468) represented the most significantly enriched group, which is important because of the role of protein phosphorylation in signaling pathways. The “nitrogen compound metabolic process” (GO:0006807) ontology was only significantly enriched in DH45 (*p* = 0.024) (Table 4). These significantly enriched biological process ontologies may reflect the mechanisms responsible for the improvement of NUE in DH45.

KEGG pathway analysis was employed for further functional categorization of the specific DEG_HP_. Nineteen KEGG pathways were significantly enriched in DH45, with the MAPK signaling pathway-plant (Ko04016) the most significantly enriched (Table 5). Further, the alanine, aspartate, and glutamate metabolism (Ko00250) and the arginine biosynthesis (Ko00220) pathways were enriched in DH45. It was noteworthy that three DEGs (*HORVU3Hr1G066090*, *HORVU4Hr1G007610,* and *HORVU4Hr1G066860*) in DH45 were mapped to nitrogen metabolism (Ko00910) and seven DEGs were mapped to alanine, aspartate, and glutamate metabolism (Ko00250), including *HORVU4Hr1G061100* (glutamate dehydrogenase), *HORVU0Hr1G017370* (aspartate aminotransferase 5), *HORVU3Hr1G073220* (aspartate aminotransferase 3), *HORVU6Hr1G003470* (aspartate aminotransferase 1), and *HORVU4Hr1G054060* (arginosuccinate lyase), as well as two that also mapped to nitrogen metabolism (*HORVU4Hr1G066860* and *HORVU4Hr1G007610*). 

### 2.7. Expression Profiles of Eight Specific DEGHP Involved in Nitrogen Metabolism 

**Figure 6:**
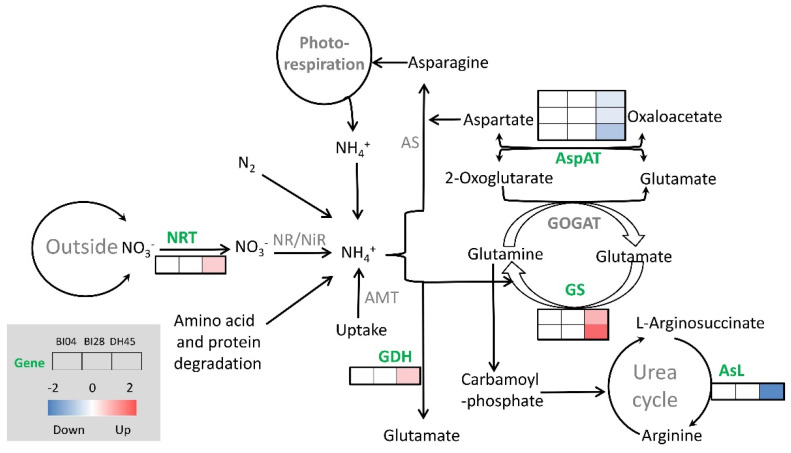
Schematic representing part of the nitrogen metabolism pathway. Relative expression levels of genes encoding specific enzymes between CK and LN treatments are shown in a color gradient from low (blue) to high (red). The columns for each heatmap are listed in order of BI04, BI28, and DH45.

Based on the significantly enriched pathways, we identified eight DEG_HP_ encoding key transporters and enzymes involved in nitrogen metabolism (Figure 6). The expression of the high-affinity nitrate transporter NRT2.5 gene (*HORVU3Hr1G066090*) and glutamate dehydrogenase (GDH) gene (*HORVU4Hr1G061100*) was up-regulated in DH45 but showed no change in the parents. Three aspartate aminotransferase (AspAT) genes (*HORVU3Hr1G073220*, *HORVU0Hr1G017370,* and *HORVU6Hr1G003470*), which are involved in the transamination of oxaloacetate to aspartate, also showed no significant difference in the parents, while their expression in DH45 was down-regulated. It was also interesting that the DEGs *HORVU4Hr1G066860* and *HORVU4Hr1G007610*, encoding glutamine synthetase (GS) enzymes HvGS1_2 and HvGS1_3, were both highly up-regulated in DH45 but showed no significant change in the parental lines. In addition, another gene encoding arginosuccinate lyase *(HORVU4Hr1G054060)* was down-regulated in DH45 but unchanged in the parents. 

### 2.8. Validation of the Expression Profiles of Eight DEG_HP_ Involved in Nitrogen Metabolism by qRT-PCR

**Figure 7:**
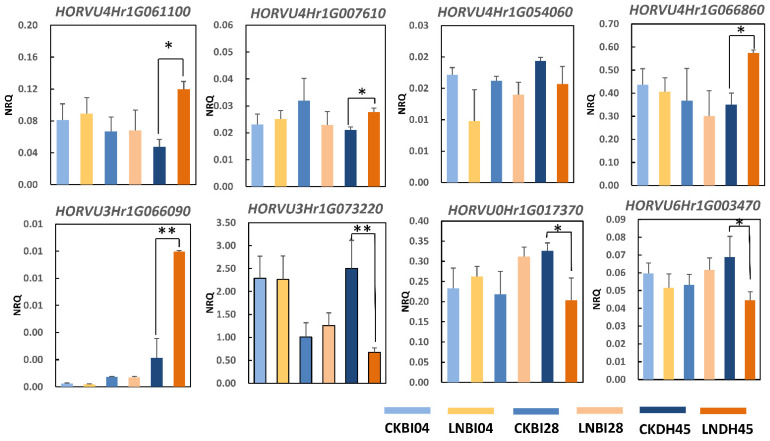
Validation of eight DEG_HP_ by quantitative real-time PCR. The X axes represent the barley samples from the parents (BI04 and BI28) and their offspring (DH45) under CK conditions and low nitrogen treatment, and the y axes represent the normalized relative quantity of gene expression. The normalized relative quantity (NRQ) is calculated as a target gene’s expression with respect to two endogenous reference genes: *HvGAPDH* and *HvActin*. * indicate significant differences at 0.05 levels by Student’s *t* test. All data represent three biological replicates. ** indicate significant differences at 0.01 levels by Student’s *t* test

**Figure 8:**
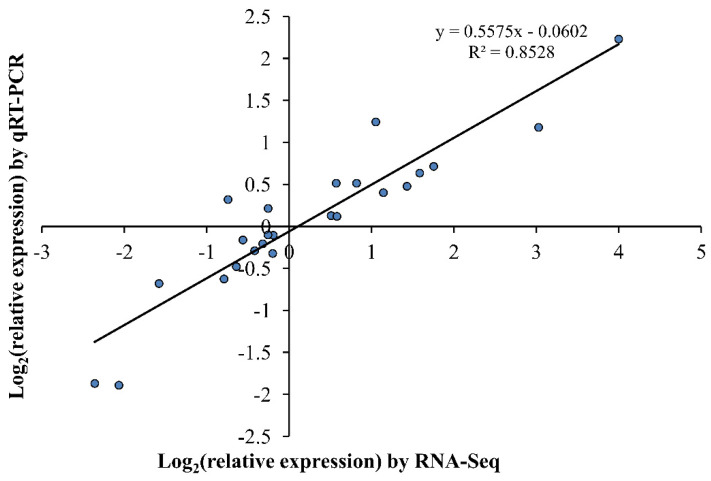
Correlation analysis of the eight DEG_HP_ for BI04, BI28, and DH45 under CK vs. LN conditions by qRT-PCR and RNA-Seq. The Pearson correlation coefficient (R^2^) was 0.8528.

Expressional levels of the eight genes (*HORVU3Hr1G066090*, *HORVU4Hr1G061100, HORVU3Hr1G073220*, *HORVU0Hr1G017370*, *HORVU6Hr1G003470*, *HORVU4Hr1G066860*, *HORVU4Hr1G007610*, and *HORVU4Hr1G054060*) involved in nitrogen metabolism were further confirmed by quantitative RT-PCR (Figure 7). The coefficient (R^2^ = 0.8528) indicated a good concordance between the data of qRT-PCR and RNA-seq, which supported the reliability of our transcriptome data (Figure 8). Genes *HORVU4Hr1G061100*, *HORVU4Hr1G007610*, *HORVU4Hr1G066860*, and *HORVU3Hr1G066090* were up-regulated while genes *HORVU3Hr1G073220*, *HORVU0Hr1G017370*, and *HORVU6Hr1G003470* were down-regulated in DH45 under LN condition compared with the CK condition. The expression of the eighth gene (*HORVU4Hr1G054060*) also showed down-regulation in DH45, but this was not significant. None of these eight genes showed a significant change in either parental line. 

### 2.9. GS and GDH Activities of DH45 and Its Parents

**Figure 9:**
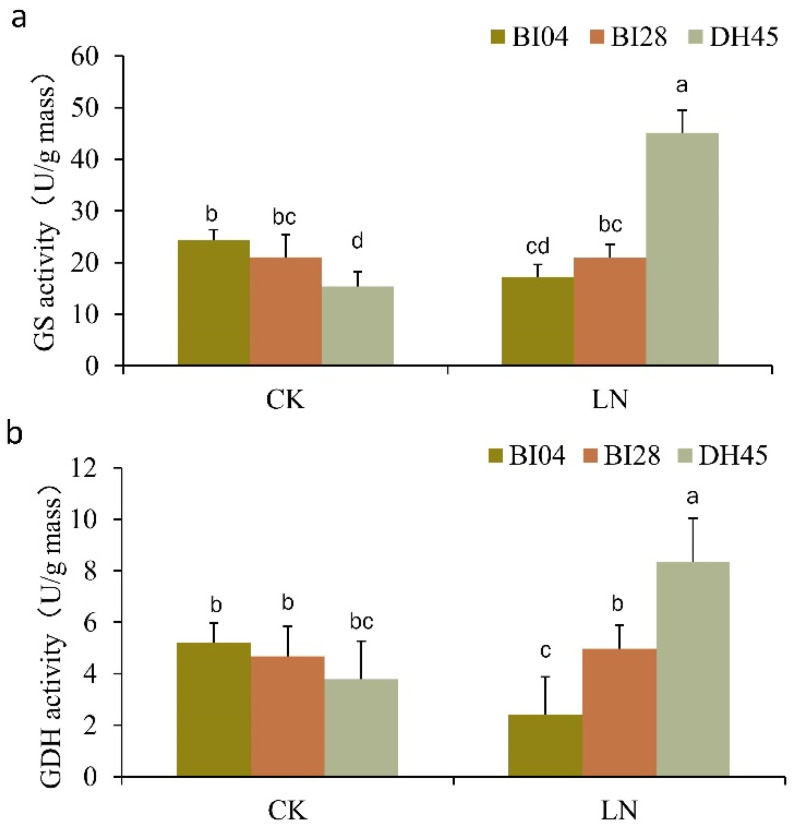
Activity of two enzymes in the leaves of DH45 and its parents after 7d of LN treatment. (**a**). GS activity; (**b**). GDH activity. Different lowercase letters indicate significant differences (*p* < 0.05) between DH45 and parents by Fisher’s least significant difference (LSD) test. The error bars are shown from the analysis of five biological replicates.

Based on the up-regulated expression patterns of the two genes encoding GS (*HORVU4Hr1G007610* and *HORVU4Hr1G066860*) and the gene encoding GDH (*HORVU4Hr1G061100*), we measured the enzyme activity of GS and GDH in 7-day-old seedlings of DH45 and its parents after LN treatment (Figure 9a,b). Under the CK conditions, the GS activity was lower in DH45 compared to its parents, while GDH activity showed no significant difference with the parents. However, the activities of both GS and GDH increased in DH45 under LN treatment and was significantly higher than that of the parents (Figure 9a,b).

## 3. Discussion

### 3.1. Production of Homozygous Lines for NUE Improvement by Crossing Combined with F1 Microspore Embryogenesis

Isolated microspore culture is a more efficient way to produce homozygous DH lines [19]. Several studies have shown that microspore mutagenesis could rapidly generate stable homozygous mutants, such as in barley [10] and *Brassica napus* [20]. In this study, a superior DH line (named DH45) with improved NUE compared with its parents was produced from in vitro culture of isolated microspores from F1 hybrids. The grain yield and NUE data collected from three continuous growing seasons showed that the performance of DH45 was stable and superior to both parents, especially under LN conditions. The results suggest that DH technologies could be applied to rapidly fix superior traits, including improved NUE, and generate new germplasm in a completely homozygous state. Our study therefore provides an alternative strategy for NUE improvement in future crop breeding. 

### 3.2. Biological Response to Low-Nitrogen Stress in the Superior Line DH45

The mechanisms involved in the response to nitrogen limitation in plants are complex and involve many pathways [21]. Numerous studies have indicated that the nitrogen compound metabolic process (GO:0006807) is associated with the utilization of nitrogen [22], and GO enrichment analysis of the selected DEG_S_ in this study showed this biological process ontology to be specifically enriched in DH45. The data suggested that nitrogen metabolism-related enzymes and transporters may be stimulated for the improvement of NUE in DH45 under nitrogen-limited conditions. Consistent with this, a study of a superior hybrid maize showed an enhancement in the nitrogen compound metabolic process to cope with low-nitrogen stress [23], while transcriptome and co-expression network analyses of *Brassica juncea* L also indicated that the nitrogen compound metabolic process could contribute to nitrogen use efficiency [24].

Nitrogen metabolism is an essential metabolic process, and other significantly enriched pathways may identify processes that have a relationship with it. KEGG enrichment analysis showed that the MAPK signaling pathway (Ko04016) was the most enriched in DH45 (Table 5). It has previously been shown that the MAPK signaling cascade plays an important role in the regulation of nitrogen assimilation in the green alga, *Chlamydomonas reinhardtii* [25]. A systematic search in Arabidopsis has also shown MAPK cascade involvement in regulating nitrogen metabolism, including the expression of nitrate reductase gene, NR2 [26]. MAPK6, specifically, has been shown to regulate NR to cause an increase in NO production [27], while MAPKKK8 is involved in the glutamate signal that brings about a change in root architecture [28]. 

In this study, we also identified that the pathways of alanine, aspartate, and glutamate metabolism (Ko00250) and arginine biosynthesis (Ko00220) were specifically enriched in DH45. In plants, arginine may be used as a nitrogen storage molecule due to its high N:C ratio (4:6) [29]. There is also increasing evidence that arginine accumulation may be a marker of stress [29,30]. Other studies have shown that the process of amino acid metabolism plays vital roles in nitrogen assimilate supply for sustainable growth in plants [31,32].

### 3.3. Exploration of Genes Potentially Responsible for NUE Improvement in the Superior Line DH45 

Previous studies have shown that differentially expressed genes between genotypes may contribute to differences in NUE [21,33]. In this study, several genes related to nitrogen metabolism were identified that were differentially expressed in DH45 but not in the parental genotypes. These became candidate genes for imparting the high NUE improvement in DH45 under LN conditions. It has been proposed that high NUE is closely linked with efficient nitrate uptake transporters [34]. Here, a gene (*HORVU3Hr1G066090*) encoding a high-affinity nitrate transporter, NRT2.5, was significantly up-regulated in DH45. It has also been reported that plants develop high-affinity nitrate transport systems (HATS) to ensure the efficiency of the influx of nitrate into roots, especially in nitrogen-limited conditions [35]. NRT2.5 acts as the most abundant transcript among the seven NRT2 family members in shoots and roots of Arabidopsis after long-term nitrogen starvation. The major role of NRT2.5 is to ensure the efficient uptake of nitrate and loading into the phloem during nitrate remobilization to maintain the growth of nitrogen-starved plants [36]. Several studies have also reported that a phosphorylation-dependent allosteric negative feedback mechanism of ammonium transporters exists to prevent excess ammonium accumulation in plants [37]. We concluded that interactions and feedback mechanisms may exist in the nutrient absorption of DH45, which favor nitrate over ammonium under nitrogen stress.

Following nitrogen uptake, glutamine synthetase (GS) and glutamate dehydrogenase (GDH) are two important enzymes of ammonium assimilation [38]. Our results showed the activities of GS and GDH in DH45 increased and were significantly higher than those of the parents under LN treatment. Two genes encoding GS (*HORVU4Hr1G066860* and *HORVU4Hr1G007610*) and a gene encoding GDH (*HORVU4Hr1G061100*) were significantly up-regulated in DH45. GS is the major route facilitating the incorporation of inorganic nitrogen into organic nitrogen, in conjunction with glutamate synthase (GOGAT). Consequently, the modification of GS is widely used as a strategy for the bioengineering of NUE [38]. It has also been reported that GDH can incorporate ammonium into glutamine in response to high levels of ammonium under stress. Glutamine and glutamate act as amino group donors in the synthesis of other amino acids required for nitrogen transport, as well as the synthesis of structural and storage proteins [37]. It awaits further investigation into the roles of GS and GDH to enhance NUE in DH45. In addition, the expression level of three DEGs (*HORVU3Hr1G073220*, *HORVU0Hr1G017370,* and *HORVU6Hr1G003470)* encoding aspartate aminotransferase (AspAT) were down-regulated in DH45 relative to the parents. Transamination of glutamate with oxaloacetate by AspAT leads to the formation of aspartate, which serves as a substrate for asparagine synthesis [39]. It was suggested that aspartate synthesis may be inhibited in DH45 to cope with low-nitrogen stress. 

## 4. Materials and Methods 

### 4.1. Plant Materials 

BI04, BI28, BI35, and BI45 are four barley varieties cultivated in the Yangtze River Delta, China. BI04 was reported previously to have good tolerance to low nitrogen [40,41], and BI28 was selected as another cultivar with good tolerance to low nitrogen. BI04 (the maternal parent) and BI28 (the paternal parent) were crossed, and microspores were isolated from the F_1_ hybrids. 

### 4.2. Isolated Microspore Culture

The microspores were isolated following the procedures described by Lu et al. [42]. Briefly, the collected spikes were subjected to cold pretreatment at 4 °C for 15 days, and then the sterilised spikes were blended in 15 mL extraction buffer using an ultra-speed blender. The extract buffer comprised 330 mM mannitol, 10 mM calcium chloride (CaCl_2_), and 5 mM MES hydrate (Sigma–Aldrich, Burlington, MA, USA). Microspores were collected by centrifugation three times, each at 100× *g* for 5 min. Then, the collected microspores were adjusted to an average density of 1.1×10^5^ mL^–1^ and placed in the dark at 25 °C for 2 days. The N6 basal medium supplemented with 2.0 µM 2.4-D, 2.3 µM KT, and 0.25 M maltose were used for embryogenic callus induction. After 21 days of culture, the induced embryogenic calli were transferred to a differentiation medium for plant regeneration. The differentiation medium was based on 9.5 mM agar solidified MS and supplemented with 2.2 µM 6-BA, 7.0 µM KT, 0.3 µM NAA, and 88 mM maltose. The conditions for in vitro plant growth are at 25 °C under the light of 150 μmol m^−2^ s^−1^ with a 16 h photoperiod. 

The ploidy of the regenerated plantlets was determined by flow cytometry. A piece of leaf approximately 0.5~1 cm was cut from a leaf blade of an individual accession and placed in a 1.5 mL EP tube. One milliliter of liquid nitrogen was added to the tube, and both were finely ground. Approximately 0.5 mL of the buffer solution (15 mmol·L^−1^ Tris, 2 mmol·L^−1^ Na_2_EDTA, 80 mmol·L^−1^ KCl, 20 mmol·L^−1^ NaCl, 0.1% Trixon X-100) was added to the tissue in the tube. This solution was resuspended several times with a pipette and then poured into a 1.5 mL new tube through a 37.5 μm filter to remove the debris. The tubes were maintained in crushed ice for at least 5 min, after which 0.2 mL of a commercial PI/RNase staining buffer solution (BD-Pharmingen) staining buffer was added to the filtered buffer solution containing the nuclei and the solution was resuspended. The samples remained on ice in the dark for 30 min before analysis. Samples were analyzed with a BD Accuri C6 flow cytometer (BD Biosciences, Franklin Lakes, NJ, USA) and BD Accuri C6 Software was used for the statistical analysis. The position of DNA peak in mesophyll cells of different ploidy plants in control group was detected as a standard to identify the ploidy level of barley later regenerated plants. Finally, the roots of haploids were treated with 0.1% colchicine for chromosome doubling. The doubled haploid (DH) plants were transferred to Kunming (Yunnan province, China), and the seeds of single plants were harvested to produce DH lines.

### 4.3. Field and Hydroponic Culture Screening

Field experiments were conducted on the farm of Shanghai Academy of Agricultural Sciences, China. Two nitrogen regimes (150 kg ha^−1^ and 45 kg ha^−1^) were applied to screen the DH lines in the growing seasons of 2016–2019. The details of soil type and nitrogen application were as described previously [15]. Relative values of grain yield were calculated as grain yield under the low nitrogen (LN) condition/grain yield under the control condition (CK). The hydroponics experiments were conducted in the laboratory of Shanghai Academy of Agricultural Sciences, China. Healthy seeds were rinsed in tap water and germinated in a phytotron. The uniform seedlings were transferred into a modified Hoagland solution for 7d and treated with two levels of nitrogen (7.5 mM and 0.75 mM). The components and treatments of the nutrient solution were the same as described previously [41]. Plants were grown under a 16 h/8 h light/dark cycle at 20 ± 2 °C with 70% relative humidity and approximately 1000 mol m^−2^ s^−1^ light intensity [43]. The solutions were replaced every 2 days and the pH was adjusted to 6.2 ± 0.2. The relative value of biomass was calculated as biomass under the LN condition/biomass under the CK condition. The relative value of NUE was calculated as NUE under the LN condition and NUE under the CK condition. 

### 4.4. Measurement of Biomass and NUE

Seedlings were collected for the determination of biomass after 7 days and 28 days of LN treatment in hydroponic culture. Fresh samples were immediately heated in an oven at 105 °C for 30 min and dried at 70 °C for 4 days. The NUE at seedling stage was calculated as the biomass relative to nitrogen supply, as described by De Macale et al. [44], and NUE at the maturity stage was defined as the grain production per unit of supplied nitrogen, according to Moll et al. [45] and Gao et al. [15]. Over middle parent value (MPD; %) and over best parent value (HPD; %) were calculated as measures of heterosis [46] using the following formulas separately: MPD = (DH−MP)/MP; and HPD = (DH−HP)/HP, where DH is the performance of a tested DH line, MP is the average performance of the two parents, and HP is the better performance of the two parents. Hypothesis testing was performed using the Student’s *t*-test. 

### 4.5. RNA Sampling, Sequencing, and Reads Mapping

The seedlings were collected after 7d of hydroponic culture with low and CK nitrogen supply for RNA isolation. Three individuals were pooled as one biological replicate. In total, 18 RNA samples from 3 materials (BI04, BI28, and DH45) under 2 treatments (CK and LN) with 3 biological replicates were prepared for RNA sequencing. All the RNA-seq was performed at the Shanghai Personalbio Biotechnology Co., Ltd. In brief, total RNA was isolated using the Trizol Reagent (Invitrogen, Thermo Fisher Scientific, Waltham, MA, USA), and the quality and integrity were determined using a Nano Drop spectrophotometer (Thermo Fisher Scientific, Waltham, MA, USA). The RNA (3 μg) was used for the construction of cDNA libraries using the TruSeq RNA Sample Preparation Kit (Illumina, San Diego, CA, USA), and nucleotide sequence data were acquired on a Hiseq platform (Illumina). Raw reads were filtered to obtain high-quality data by removing adapters and excluding low-quality reads with Q < 20 or ambiguous bases (‘N’). The resulting clean reads were mapped onto the reference genome of barley (http://plants.ensembl.org/Hordeum_vulgare/Info/Index?db=core), according to the reference [47]. Cutadapt (https://cutadapt.readthedocs.io/en/stable/) was used for trimming the adapters and Tophat2 (http://ccb.jhu.edu/software/tophat/index.shtml) for mapping the raw reads to the barley genome. The datasets generated and analyzed during the current study are available in the National Center for Biotechnology Information: Submission ID: SUB6990379; Bio Project ID: PRJNA607428.

### 4.6. Identification of DEGs and Functional Analysis

The methods for identification of DEGs and functional analysis were adapted from those described previously [43]. Differential expression analysis was performed using the R package DESeq. For all comparisons, the resulting *p*-values were adjusted using Benjamini and Hochberg’s approach for controlling the false-discovery rate. Genes with [Log_2_ fold change] > 1 and a statistical *p*-value < 0.05 were classed as significantly differentially expressed genes (DEGs). Venn diagram software (http://bioinfogp.cnb.csic.es/tools/venny/index.html) was used to sort the DEGs, and R language pheatmap software was used for hierarchical cluster analysis of genes differentially expressed between the hybrid and parents (DEG_HP_) after normalization. The enrichment analysis of GO and KEGG pathways were performed to assign possible functional categorization and metabolic pathways using AgriGO tool (http://systemsbiology.cau.edu.cn/agriGOv2/). 

### 4.7. Validation of RNA-Seq by qRT-PCR

To validate the RNA-Seq data, 1 μg total RNA of each sample was used for preparation of a template for a qRT-PCR assay. After DNase digestion, first strand cDNA was synthesized according to the manufacturer’s recommended procedures (Invitrogen, Thermo Fisher Scientific, Tokyo, Japan). Real-time PCR was performed in a F7500 Fast system (Applied Biosystems, Waltham, MA, USA) using 2 × power-up SYBR qPCR Mix (Toyobo). The PCR conditions and data analysis were conducted as described [48]. Expression was represented as the normalized relative quantity (NRQ) of a target gene’s expression with respect to two endogenous reference genes: *HvGAPDH* and *HvActin*. Primer pairs were designed using primer3 software (http:/www.ncbi.nlm.nih.gov/tools/primerblast/), and all the primers are presented in Table S4. Amplification efficiencies were evaluated by the LinRegPCR software. 

### 4.8. Quantification of GS and GDH Activities

The activities of GS and GDH were determined after 7 days of LN supply using protocol (CAT No. BC0915 and CAT No. BC1460) of the Solarbio company, China. Briefly, 100 mg fresh leaves were ground with 1 mL extract buffer of GS and GDH in an ice bath. The resulting homogenates were centrifuged for 10 min at 4000× *g* at 4 °C and the supernatants collected to be used as the crude enzyme extract. GS and GDH activities were measured by spectrophotometer at 540 nm and 340 nm, respectively. 

### 4.9. Statistical Analysis

Unless specified otherwise, comparisons between different treatments were tested by the least significant difference (LSD) test using SPSS 21.0 statistical software, and the difference at 0.05 level (*p* < 0.05) was considered as statistically significant.

## 5. Conclusions

A homozygous barley DH line (named DH45) which was produced by crossing combined with microspore technologies, exhibited better performance on biomass, grain yield and NUE over its parents under LN conditions. Transcriptomic profiling revealed an enriched nitrogen metabolism pathway and identified several candidate genes that could contribute to the superior NUE in line DH45. Taken together, our work partially explored the mechanism of NUE improvement in the superior DH line and exhibited the potential to fix superior traits via conventional crossing combined with microspore culture in future crop breeding. Our results reveal that the combination of crossing with F_1_ microspore culture is an efficient approach to rapidly generate DH lines for the improvement of NUE. 

## Figures and Tables

**Table 1:** The comparison of NUE in four genotypes of barley seedlings grown under two nitrogen regimes, LN and CK.

Genotype	NUE/(g.g^−1^)		Relative Value of NUE
	Control (CK)	Low Nitrogen (LN)	
BI-04	23.95 ± 0.83 ^ab^	45.8 ± 4.16 ^a^	1.91
BI-28	24.49 ± 0.09 ^a^	45.27 ± 0.72 ^ab^	1.84
BI-35	23.34 ± 0.45 ^b^	41.17 ± 1.53 ^bc^	1.76
BI-45	23.05 ± 0.34 ^b^	37.83 ± 1.20 ^c^	1.64

NUE (g.g^−1^) is defined as the grain production (g) per unit of supplied N (g). Different lowercase letters within the table indicate significant differences at 0.05 levels by Fisher’s least significant difference (LSD) test. All data represent three biological replicates.

**Table 2:** Comparison of grain yield and NUE between DH45 and parents at maturity stage (Zadoks growth scale 91) in field experiments (2016–2019).

Treatment and Line		2016–2017		2017–2018		2018–2019	
		GY/g	NUE/g·g^−1^	GY/g	NUE/g·g^−1^	GY/g	NUE/g·g^−1^
Control (CK)	BI04	14.5 ± 1.53 ^ab^	46.24 ± 4.88 ^d^	8.03 ± 2.55 ^ab^	25.61 ± 8.13 ^c^	20.4 ± 0.51 ^a^	65.05 ± 13.72 ^b^
	BI28	16.08 ± 2.16 ^a^	51.27 ± 6.88 ^cd^	9.59 ± 2.38 ^a^	30.57 ± 7.60 ^c^	16.27 ± 0.72 ^a^	51.87 ± 6.92 ^b^
	DH45	14.24 ± 2.58 ^ab^	45.4 ± 8.22 ^d^	9.64 ± 1.69 ^a^	30.73 ± 5.40 ^c^	18.84 ± 0.86 ^a^	60.06 ± 12.43 ^b^
Low nitrogen (LN)	BI04	6.11 ± 1.72 ^d^	69.26 ± 19.45 ^c^	4.17 ± 1.60 ^c^	47.27 ± 10.16 ^b^	6.34 ± 0.57 ^c^	71.86 ± 15.61 ^b^
	BI28	10.43 ± 1.60 ^c^	118.23 ± 18.08 ^b^	4.24 ± 0.95 ^c^	48.06 ± 10.79 ^b^	5.52 ± 0.53 ^c^	62.57 ± 12.27 ^b^
	DH45	13.22 ± 1.86 ^b^	149.86 ± 21.09 ^a^	5.67 ± 1.41 ^bc^	64.27 ± 16.03 ^a^	10.00 ± 0.62 ^b^	113.36 ± 26.41 ^a^

GY(g): grain yield (g); NUE(g·g^−1^): grain production (g) per unit of supplied N (g); Different lowercase letters within the same column indicate significant differences at 0.05 levels by Fisher’s least significant difference (LSD) test. All data represent four biological replicates.

**Table 3:** Comparison of shoot biomass and NUE between DH45 and parents at seedling growth stage (Zadoks growth scale 19) in hydroponic culture.

Traits	Nitrogen Regimes	Mean ± SD			MPD (%)	HPD (%)
		BI04	BI28	DH45		
Biomass/g	Control (CK)	0.35±0.02 ^a^	0.36±0.03 ^a^	0.39±0.03 ^a^	8.97	7.69
	Low nitrogen (LN)	0.14±0.02 ^c^	0.16±0.02 ^c^	0.27±0.01 ^b^	80.00 *	68.75 *
NUE/g·g^-1^	Control (CK)	7.92±1.72 ^C^	8.32±1.53 ^C^	9.00±1.63 ^C^	10.84	8.17
	Low nitrogen (LN)	32.00±1.74 ^B^	37.23±1.63 ^B^	61.31±1.33 ^A^	77.11 *	64.68 *

NUE(g·g^−1^) was defined as the biomass (g) relative to nitrogen supply (g); MPD: over middle parent value; HPD: over best parent value. Different lowercase and capital letters indicate significant differences at 0.05 level by Fisher’s least significant difference (LSD) test within the biomass and NUE under CK (7.5 mM) and LN (0.75 mM) conditions, respectively. * indicates significant differences at 0.05 levels by Student’s *t* test. All data represent three biological replicates.

**Table 4:** Biological process terms of GO enrichment for 1027 DEG_HP_ in DH45.

Term ID	Description	−Log_10_ (*p* Value)		
		BI04	BI28	DH45
GO:0006468	protein phosphorylation	2.91	6.14	9.09
GO:0048544	recognition of pollen			7.23
GO:0015696	ammonium transport			3.46
GO:0005975	carbohydrate metabolic process		5.72	2.22
GO:0006355	regulation of transcription, DNA-templated		10.99	2.18
GO:0006644	phospholipid metabolic process			1.67
GO:0006665	sphingolipid metabolic process			1.67
GO:0006807	nitrogen compound metabolic process			1.61
GO:0016042	lipid catabolic process			1.46
GO:0006817	phosphate ion transport			1.37

BI04, BI28, and DH45 represents the DEGs under the CKBI04 vs. LN BI04 condition, CKBI28 vs. LNBI28 condition, and the selected 1027 DEG_HP_, respectively. The blank without values means that there are no DEGs to be assigned in the listed pathways. Only categories with *p* < 0.05 are displayed.

**Table 5:** Metabolic pathways of KEGG enrichment for selected 1027 DEG_HP_ in DH45 relative to the parents.

Pathway ID	Description	−Log_10_ (*p* Value)		
		BI04	BI28	DH45
ko04016	MAPK signaling pathway-plant		1.66	4.04
ko00250	Alanine, aspartate and glutamate metabolism			3.97
ko00052	Galactose metabolism		1.95	3.55
ko00220	Arginine biosynthesis			3.18
ko00910	Nitrogen metabolism	0.81		2.59
ko00940	Phenylpropanoid biosynthesis			2.58
ko00330	Arginine and proline metabolism			2.48
ko04014	Ras signaling pathway		0.75	2.48
ko00500	Starch and sucrose metabolism		1.95	2.40
ko04075	Plant hormone signal transduction		1.31	2.13
ko00360	Phenylalanine metabolism			2.11
ko04010	MAPK signaling pathway			1.96
ko04141	Protein processing in endoplasmic reticulum		1.05	1.85
ko00592	Alpha-Linolenic acid metabolism		0.66	1.70
ko00511	Other glycan degradation			1.50
ko00565	Ether lipid metabolism			1.47
ko00270	Cysteine and methionine metabolism		1.16	1.44
ko00590	Arachidonic acid metabolism		0.94	1.43
ko00564	Glycerophospholipid metabolism			1.35

BI04, BI28, and DH45 represents the DEGs under the CKBI04 vs. LN BI04 condition, CKBI28 vs. LNBI28 condition, and the selected 1027 DEG_HP_, respectively. The blank without values means that there are no DEGs to be assigned in the listed pathways. Only categories with *p* < 0.05 are displayed.

## Data Availability

The datasets generated and analyzed during the current study are available through the National Center for Biotechnology Information: Submission ID: SUB6990379; Bio Project ID: PRJNA607428.

## Supplementary Materials

The following are available online at https://www.mdpi.com/article/10.3390/plants10081588/s1, Table S1: RNA integrity number of samples used for sequencing. Table S2: Summary of quality control for RNA-Seq data. Table S3: Summary of mapped events for clean reads. Table S4: Primer sequences for qRT-PCR assay. Figure S1: Principal component analysis (PCA) analysis of the samples based on gene counts. Figure S2: Up-and down-regulated differentially expression genes betweenDH45 and parents.

## Abbreviations

CK	Control
LN	Low nitrogen
GY	Grain yield per plant
NUE	Nitrogen use efficiency
DH	Doubled haploid
HPD	Over best parent value
MPD	Over middle parent value
GO	Gene Ontology
KEGG	Kyoto Encyclopedia of Genes and Genomes
DEGs	Differentially expressed genes
NRQ	Normalized relative quantity
