# Supplementary material for: Rapid Generation and Analysis of a Barley Doubled Haploid Line with Higher Nitrogen Use Efficiency Than Parental Lines by F1 Microspore Embryogenesis
Source: Plants (Basel). 2021 Aug 1;10(8):1588. doi: 10.3390/plants10081588 (PMC8401716; doi:10.3390/plants10081588)
Supplement: Supplementary file 1 [file plants-10-01588-s001.zip › plants-1305821-supplementary.pdf]

Table S1: RNA integrity number of samples used for sequencing

Name	Total quantity (μg)	OD260/280	OD260/230	RIN value
CK BI04-1	4.285	1.978	1.824	7.5
CK BI04-2	4.108	2.055	1.852	6.9
CK BI04-3	4.990	2.072	1.891	7.7
CK BI28-1	5.471	2.066	2.157	7.2
CK BI28-2	5.911	2.071	1.926	7.2
CK BI28-3	5.018	2.074	1.853	6.9
CK DH45-1	13.553	2.100	1.815	6.8
CK DH45-2	7.114	2.078	2.205	7.2
CK DH45-3	4.787	2.025	1.838	7.4
LN BI04-1	5.182	2.089	1.891	7.5
LN BI04-2	3.958	2.048	1.826	7.1
LN BI04-3	6.409	2.073	1.876	6.9
LN BI28-1	9.298	2.086	2.196	8.4
LN BI28-2	5.209	2.064	2.114	7.4
LN BI28-3	3.217	2.031	1.879	7.6
LN DH45-1	1.560	2.062	1.899	7.1
LN DH45-2	3.513	2.019	1.871	7.4
LN DH45-3	5.685	2.076	1.979	7.3

Table S2: Summary of quality control for RNA-Seq data

Sample	Total raw reads No.	Total clean reads No.	N(%)	Q20 (%)	Q30(%)	Clean Reads %
CK BI-04	136,345,154	135,624,126	0.001613	97.02	92.74	99.47
CK BI-28	127,995,806	127,447,370	0.000959	97.35	93.46	99.57
CK DH45	132,055,610	131,105,758	0.002032	96.45	91.50	99.29
LN BI-04	130,560,670	129,487,894	0.002811	96.04	90.63	99.18
LN BI-28	121,972,182	121,122,070	0.00304	96.34	91.22	99.30
LN DH45	137,467,718	136,336,736	0.002581	96.07	90.66	99.17

CK means the N level with 7.5mmol·L^{-1}; LN means the N level with 0.75mmol·L^{-1}; Total Raw Reads: The number of reads before filtering; Total Clean Reads: Filtered reads; N (%): The percentage of ambiguous bases; Q20 (%): The percentage of bases whose recognition accuracy is above 99%; Q30 (%): The percentage of bases whose recognition accuracy is above 99.9%; Clean Reads Ratio (%): The proportion of filtered reads.

Table S3: Summary of mapped events for clean reads

Sample	Map_Events	Mapped_to_Gene	Mapped_to_InterGene	Mapped to Exon
CK BI04-1	33,888,898	32,260,807(95.20%)	1,628,091(4.80%)	31,989,644(99.16%)
CK BI04-2	35,867,807	34,236,826(95.45%)	1,630,981(4.55%)	33,980,017(99.25%)
CK BI04-3	35,526,589	33,814,162(95.18%)	1,712,427(4.82%)	33,551,714(99.22%)
CK BI28-1	34,065,923	32,335,504(94.92%)	1,730,419(5.08%)	32,069,746(99.18%)
CK BI28-2	32,189,874	30,539,458(94.87%)	1,650,416(5.13%)	30,277,962(99.14%)
CK BI28-3	32,613,411	30,829,754(94.53%)	1,783,657(5.47%)	30,536,466(99.05%)
CK DH45-1	31,369,781	30,002,095(95.64%)	1,367,686(4.36%)	29,781,854(99.27%)
CK DH45-2	33,545,207	31,994,554(95.38%)	1,550,653(4.62%)	31,758,179(99.26%)
CK DH45-3	34,537,693	32,724,680(94.75%)	1,813,013(5.25%)	32,454,572(99.17%)
LN BI04-1	30,439,496	28,962,068(95.15%)	1,477,428(4.85%)	28,707,006(99.12%)
LN BI04-2	34,571,444	32,766,357(94.78%)	1,805,087(5.22%)	32,431,898(98.98%)
LN BI04-3	32,330,526	30,505,288(94.35%)	1,825,238(5.65%)	30,172,115(98.91%)
LN BI28-1	30,767,263	29,221,279(94.98%)	1,545,984(5.02%)	28,978,494(99.17%)
LN BI28-2	30,768,681	29,250,460(95.07%)	1,518,221(4.93%)	28,972,412(99.05%)
LN BI28-3	29,072,572	27,583,938(94.88%)	1,488,634(5.12%)	27,356,699(99.18%)
LN DH45-1	33,556,455	31,915,988(95.11%)	1,640,467(4.89%)	31,698,210(99.32%)
LN DH45-2	34,878,170	33,173,687(95.11%)	1,704,483(4.89%)	32,917,244(99.23%)
LN DH45-3	33,580,138	32,051,766(95.45%)	1,528,372(4.55%)	31,854,734(99.39%)

Table S4: Primer information for qRT-PCR assay

Gene ID	Forward primer (5'-3')	Reverse primer (3'-5')
HvActin	GCTGACCGTATGAGCAAGGA	GGAAAGTGCTGAGTGAGGCT
HvGAPDH	AGATGCCCCTATGTTTGTGG	GGTCTTCTGTGTGGCAGTGA
HORVU3Hr1G066090	GGAGATGCACAAGGACAGCT	GTAGAAGTACTGCGCGACGA
HORVU4Hr1G061100	CACCAACGCATAGACAATGG	CAAACAAAACTCCCCTTCCA
HORVU3Hr1G073220	CTGCAACTCGTGGACTGGAT	AGTAGGGTTGTGGGCACATG
HORVU0Hr1G017370	GCGGATCAAGAATGTTCGGC	TCAAGCCCGTGAAGGAGAAC
HORVU6Hr1G003470	TCCCACTGAGGAACAATGGC	TGGCATCCCTCTCTGGATCA
HORVU4Hr1G054060	CATTGGCGAAGAGTGGGTCT	TTCCACGGACAAGCTCCATC
HORVU4Hr1G066860	AACCTCAACCTCAGCGACTG	GTCGTAGTTCCACTTGGGCA
HORVU4Hr1G007610	GGATGGACGTCAGGAGCAAA	AGGATGACTTCGCTGTCGTC

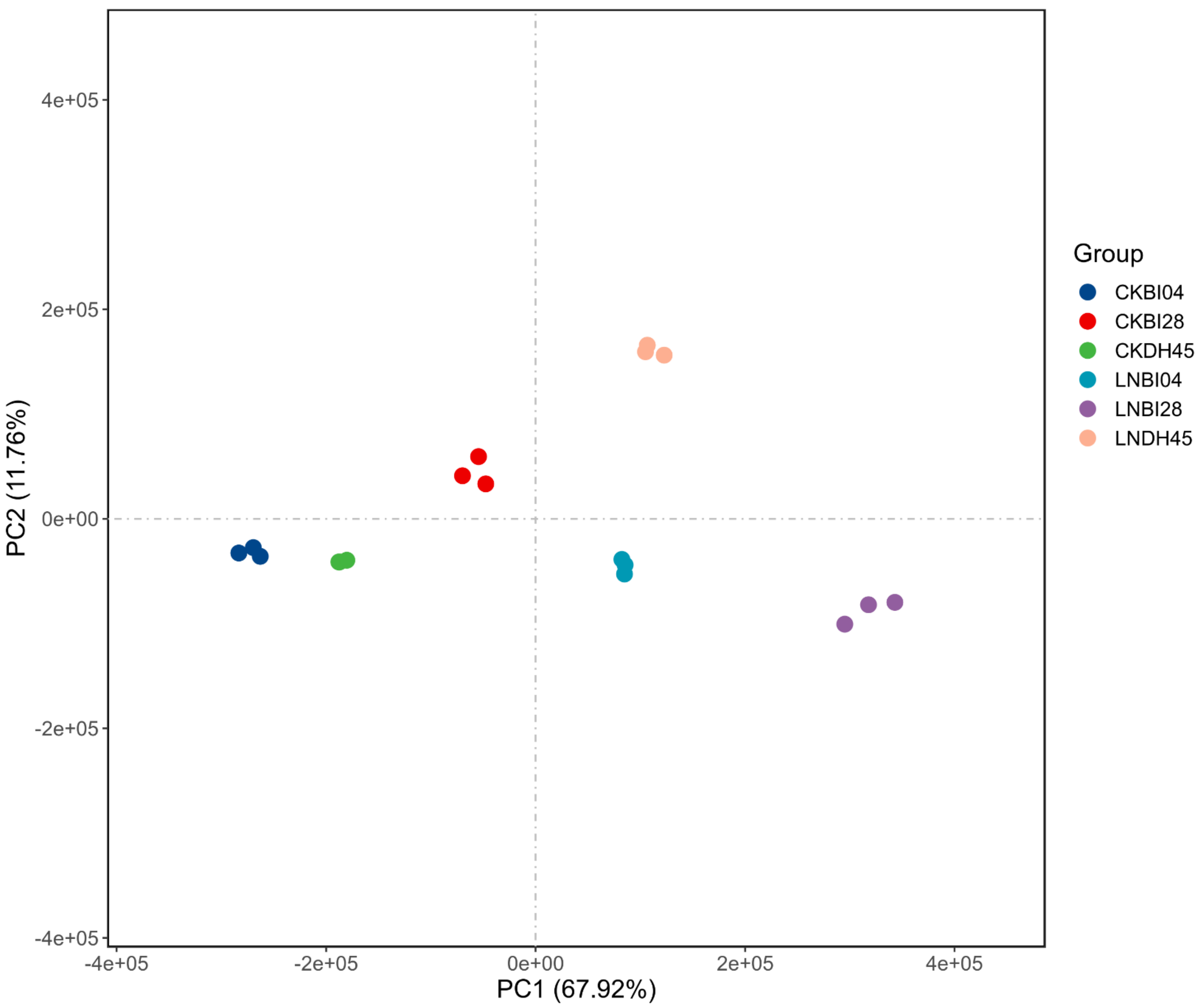

Figure S1: Principal component analysis (PCA) analysis of the samples based on gene counts

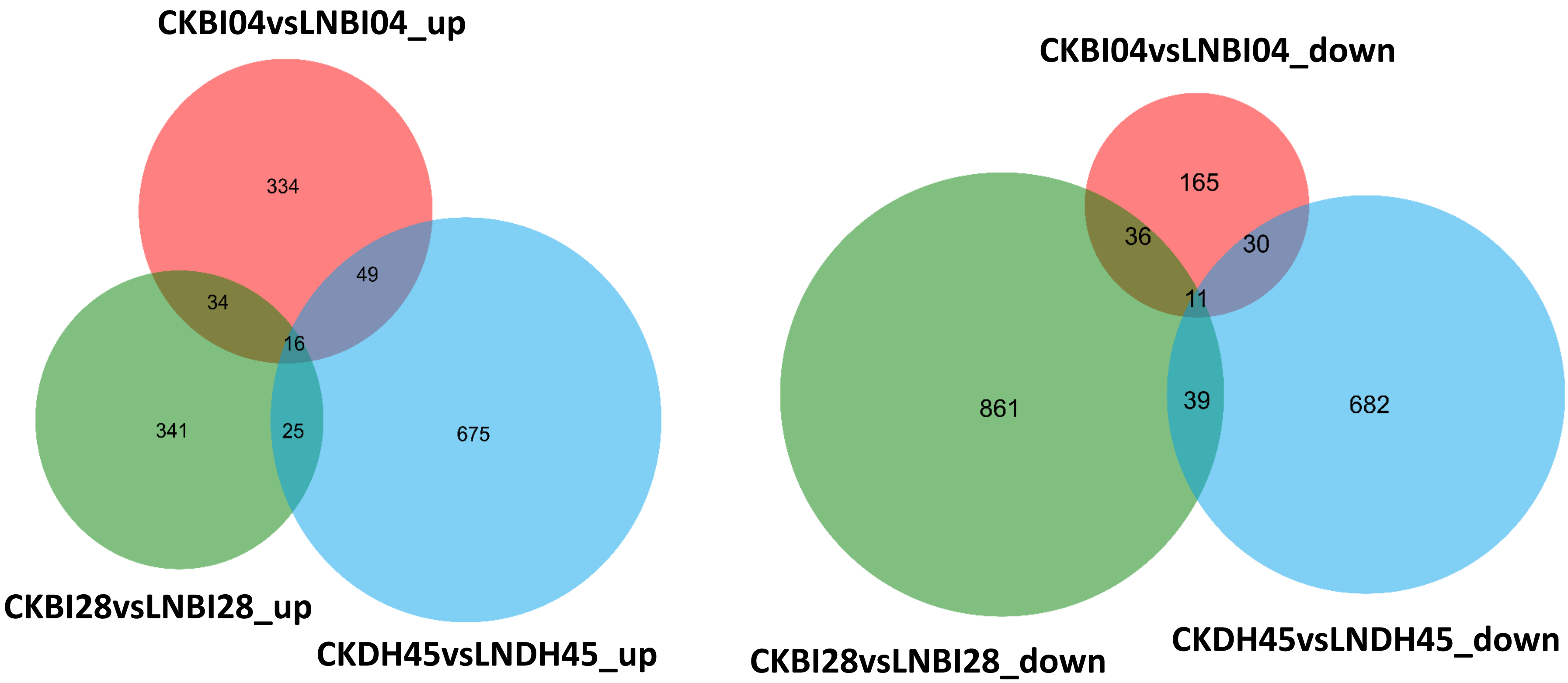

Figure S2: Up-and down-regulated differentially expression genes betweenDH45 and parents
